# Supplementary material for: Clinical Effectiveness of Restorative Materials for the Restoration of Carious Primary Teeth: An Umbrella Review
Source: J Clin Med. 2022 Jun 17;11(12):3490. doi: 10.3390/jcm11123490 (PMC9225564; doi:10.3390/jcm11123490)
Supplement: Supplementary file 1 [file jcm-11-03490-s001.zip › Supplementary Table S1_Search Strategy.pdf]

**Table S1.** Search strategy.

| Search number | Filters | Search Details | Results |
|---|---|---|---|
| 1 | | ("restorati*"[Title/Abstract] OR "crown"[Title/Abstract] OR "filling*"[Title/Abstract]) AND ("cari*"[Title/Abstract] OR "decay"[Title/Abstract] OR "cavities"[Title/Abstract] OR "dentine"[Title/Abstract] OR "lesion"[Title/Abstract]) AND (("primary"[Title/Abstract] OR "baby"[Title/Abstract] OR "deciduous"[Title/Abstract] OR "milk"[Title/Abstract]) AND ("tooth"[Title/Abstract] OR "teeth"[Title/Abstract] OR "dental"[Title/Abstract] OR "dentition"[Title/Abstract])) AND "review"[Title] | 89 |
| 2 | | ("glass"[Title/Abstract] OR "polyalkenoate"[Title/Abstract] OR "ionomer"[Title/Abstract] OR "cement*"[Title/Abstract] OR "resin*"[Title/Abstract] OR "metal"[Title/Abstract] OR "composite*"[Title/Abstract] OR "amalgam"[Title/Abstract] OR "compomer*"[Title/Abstract] OR "Polyacid"[Title/Abstract] OR "biomaterial"[Title/Abstract] OR "bio-active"[Title/Abstract]) AND (("restorati*"[Title/Abstract] OR "crown"[Title/Abstract] OR "filling*"[Title/Abstract]) AND ("cari*"[Title/Abstract] OR "decay"[Title/Abstract] OR "cavities"[Title/Abstract] OR "dentine"[Title/Abstract] OR "lesion"[Title/Abstract]) AND (("primary"[Title/Abstract] OR "baby"[Title/Abstract] OR "deciduous"[Title/Abstract] OR "milk"[Title/Abstract]) AND ("tooth"[Title/Abstract] OR "teeth"[Title/Abstract] OR "dental"[Title/Abstract] OR "dentition"[Title/Abstract]))) AND "review"[Title] | 44 |
| 3 | | "tooth, deciduous"[MeSH Terms] AND ("glass"[Title/Abstract] OR "polyalkenoate"[Title/Abstract] OR "ionomer"[Title/Abstract] OR "cement*"[Title/Abstract] OR "resin*"[Title/Abstract] OR "metal"[Title/Abstract] OR "composite*"[Title/Abstract] OR "amalgam"[Title/Abstract] OR "compomer*"[Title/Abstract] OR "Polyacid"[Title/Abstract] OR "biomaterial"[Title/Abstract] OR "bio-active"[Title/Abstract]) AND "review"[Title] | 48 |
| 4 | | "tooth, deciduous"[MeSH Terms] AND ("glass"[Title/Abstract] OR "polyalkenoate"[Title/Abstract] OR "ionomer"[Title/Abstract] OR "cement*"[Title/Abstract] OR "resin*"[Title/Abstract] OR "metal"[Title/Abstract] OR "composite*"[Title/Abstract] OR "amalgam"[Title/Abstract] OR "compomer*"[Title/Abstract] OR "Polyacid"[Title/Abstract] OR "biomaterial"[Title/Abstract] OR "bio-active"[Title/Abstract]) AND "humans"[MeSH Terms] AND (("restorati*"[Title/Abstract] OR "crown"[Title/Abstract] OR "filling*"[Title/Abstract]) AND ("cari*"[Title/Abstract] OR "decay"[Title/Abstract] OR "cavities"[Title/Abstract] OR "dentine"[Title/Abstract] OR "lesion"[Title/Abstract]) AND (("primary"[Title/Abstract] OR "baby"[Title/Abstract] OR "deciduous"[Title/Abstract] OR "milk"[Title/Abstract]) AND ("tooth"[Title/Abstract] OR "teeth"[Title/Abstract] OR "dental"[Title/Abstract] OR "dentition"[Title/Abstract]))) AND ("review"[Publication Type] OR "review literature as topic"[MeSH Terms] OR "review"[All Fields]) | 52 |
| 5 | Systematic Review | ("tooth, deciduous"[MeSH Terms] AND ("glass"[Title/Abstract] OR "polyalkenoate"[Title/Abstract] OR "ionomer"[Title/Abstract] OR "cement*"[Title/Abstract] OR "resin*"[Title/Abstract] OR "metal"[Title/Abstract] OR "composite*"[Title/Abstract] OR "amalgam"[Title/Abstract] OR "compomer*"[Title/Abstract] OR "Polyacid"[Title/Abstract] OR "biomaterial"[Title/Abstract] OR "bio-active"[Title/Abstract]) AND "humans"[MeSH Terms] AND (("restorati*"[Title/Abstract] OR "crown"[Title/Abstract] OR "filling*"[Title/Abstract]) AND ("cari*"[Title/Abstract] OR "decay"[Title/Abstract] OR "cavities"[Title/Abstract] OR "dentine"[Title/Abstract] OR "lesion"[Title/Abstract]) AND (("primary"[Title/Abstract] OR "baby"[Title/Abstract] OR "deciduous"[Title/Abstract] OR "milk"[Title/Abstract]) AND ("tooth"[Title/Abstract] OR "teeth"[Title/Abstract] OR "dental"[Title/Abstract] OR "dentition"[Title/Abstract])))) AND (systematicreview[Filter]) | 18 |
| 6 | Systematic Review | (("glass"[Title/Abstract] OR "polyalkenoate"[Title/Abstract] OR "ionomer"[Title/Abstract] OR "cement*"[Title/Abstract] OR "resin*"[Title/Abstract] OR "metal"[Title/Abstract] OR "composite*"[Title/Abstract] OR "amalgam"[Title/Abstract] OR "compomer*"[Title/Abstract] OR "Polyacid"[Title/Abstract] OR "biomaterial"[Title/Abstract] OR "bio-active"[Title/Abstract]) AND (("restorati*"[Title/Abstract] OR "crown"[Title/Abstract] OR "filling*"[Title/Abstract]) AND ("cari*"[Title/Abstract] OR "decay"[Title/Abstract] OR "cavities"[Title/Abstract] OR "dentine"[Title/Abstract] OR "lesion"[Title/Abstract]) AND (("primary"[Title/Abstract] OR "baby"[Title/Abstract] OR "deciduous"[Title/Abstract] OR "milk"[Title/Abstract]) AND ("tooth"[Title/Abstract] OR "teeth"[Title/Abstract] OR "dental"[Title/Abstract] OR "dentition"[Title/Abstract])))) AND (systematicreview[Filter]) | 40 |
| 7 | Systematic Review | (("restorati*"[Title/Abstract] OR "crown"[Title/Abstract] OR "filling*"[Title/Abstract]) AND ("cari*"[Title/Abstract] OR "decay"[Title/Abstract] OR "cavities"[Title/Abstract] OR "dentine"[Title/Abstract] OR "lesion"[Title/Abstract]) AND (("primary"[Title/Abstract] OR "baby"[Title/Abstract] OR "deciduous"[Title/Abstract] OR "milk"[Title/Abstract]) AND ("tooth"[Title/Abstract] OR "teeth"[Title/Abstract] OR "dental"[Title/Abstract] OR "dentition"[Title/Abstract]))) AND (systematicreview[Filter]) | 71 |
| 8 | Systematic Review | ((("dental materials"[MeSH Terms] AND "tooth, deciduous"[MeSH Terms]) OR "tooth, deciduous"[MeSH Terms]) AND (("restorati*"[Title/Abstract] OR "crown"[Title/Abstract] OR "filling*"[Title/Abstract]) AND ("cari*"[Title/Abstract] OR "decay"[Title/Abstract] OR "cavities"[Title/Abstract] OR "dentine"[Title/Abstract] OR "lesion"[Title/Abstract]))) AND (systematicreview[Filter]) | 29 |
| 9 | Systematic Review | (("glass"[Title/Abstract] OR "polyalkenoate"[Title/Abstract] OR "ionomer"[Title/Abstract] OR "cement*"[Title/Abstract] OR "resin*"[Title/Abstract] OR "metal"[Title/Abstract] OR "composite*"[Title/Abstract] OR "amalgam"[Title/Abstract] OR "compomer*"[Title/Abstract] OR "Polyacid"[Title/Abstract] OR "biomaterial"[Title/Abstract] OR "bio-active"[Title/Abstract]) AND (("restorati*"[Title/Abstract] OR "crown"[Title/Abstract] OR "filling*"[Title/Abstract]) AND ("cari*"[Title/Abstract] OR "decay"[Title/Abstract] OR "cavities"[Title/Abstract] OR "dentine"[Title/Abstract] OR "lesion"[Title/Abstract]) AND (("primary"[Title/Abstract] OR "baby"[Title/Abstract] OR "deciduous"[Title/Abstract] OR "milk"[Title/Abstract]) AND ("tooth"[Title/Abstract] OR "teeth"[Title/Abstract] OR "dental"[Title/Abstract] OR "dentition"[Title/Abstract]))) AND ("control*"[All Fields] OR "random*"[All Fields])) AND (systematicreview[Filter]) | 33 |
| 10 | | ("glass"[Title/Abstract] OR "polyalkenoate"[Title/Abstract] OR "ionomer"[Title/Abstract] OR "cement*"[Title/Abstract] OR "resin*"[Title/Abstract] OR "metal"[Title/Abstract] OR "composite*"[Title/Abstract] OR "amalgam"[Title/Abstract] OR "compomer*"[Title/Abstract] OR "Polyacid"[Title/Abstract] OR "biomaterial"[Title/Abstract] OR "bio-active"[Title/Abstract]) AND (("restorati*"[Title/Abstract] OR "crown"[Title/Abstract] OR "filling*"[Title/Abstract]) AND ("cari*"[Title/Abstract] OR "decay"[Title/Abstract] OR "cavities"[Title/Abstract] OR "dentine"[Title/Abstract] OR "lesion"[Title/Abstract]) AND (("primary"[Title/Abstract] OR "baby"[Title/Abstract] OR "deciduous"[Title/Abstract] OR "milk"[Title/Abstract]) AND ("tooth"[Title/Abstract] OR "teeth"[Title/Abstract] OR "dental"[Title/Abstract] OR "dentition"[Title/Abstract]))) AND ("control*"[All Fields] OR "random*"[All Fields]) AND "review"[Title] | 23 |
| 11 | | (("dental materials"[MeSH Terms] AND "tooth, deciduous"[MeSH Terms]) OR "tooth, deciduous"[MeSH Terms]) AND (("restorati*"[Title/Abstract] OR "crown"[Title/Abstract] OR "filling*"[Title/Abstract]) AND ("cari*"[Title/Abstract] OR "decay"[Title/Abstract] OR "cavities"[Title/Abstract] OR "dentine"[Title/Abstract] OR "lesion"[Title/Abstract])) AND ("control*"[All Fields] OR "random*"[All Fields]) AND "review"[Title] | 19 |
