# Supplementary material for: Clinical Effectiveness of Restorative Materials for the Restoration of Carious Primary Teeth: An Umbrella Review
Source: J Clin Med. 2022 Jun 17;11(12):3490. doi: 10.3390/jcm11123490 (PMC9225564; doi:10.3390/jcm11123490)
Supplement: Supplementary file 1 [file jcm-11-03490-s001.zip › Supplementary Tables S2 and S3.pdf]

Table S2. Reason for exclusion of reviews retrieved for the current umbrella review.

**Reasons for exclusion (for studies with more than 1 reason, only 1 reason will be given)**	**Title, Year and Authors**	**Number of papers**
Less than 12 months follow-up or follow-up not reported for initial studies	Aiem et al., 2020; Alrashdi et al., 2021; Badar et al., 2019; Dorri et al., 2017; Santos et al., 2016; Schwendicke et al., 2016.	6
Does not meet inclusion criteria (does not compare the survival of different restorative materials)	Aiem et al., 2017; de Amorim et al., 2012; de Amorim et al., 2018; Duangthip et al., 2016; Pedrotti et al., 2019.	5
Does not meet inclusion criteria (inclusion of permanent teeth)	Arbildo-Vega et al., 2020; Brunthaler et al., 2003; Demarco et al., 2015; Fron Chabouis et al., 2013; Heintze et al., 2012; Moraschini et al., 2015; Opdam et al., 2014; Paula et al., 2019; Szesz et al., 2017; Worthington et al., 2021.	10
Does not meet inclusion criteria (inclusion of in vitro studies)	Lenzi et al., 2016.	1
Wrong outcome (does not evaluate restoration failure in dependence to the chosen restorative material)	Coll et al., 2020; do Amaral et al., 2016; Marquillier et al., 2018; Montedori et al., 2016; Raggio et al., 2016; Ricketts et al., 2013; Schwendicke et al., 2013; Schwendicke et al., 2021; van der Sande et al., 2013; Virupaxi et al., 2020; Wakhloo et al., 2021.	11
Wrong publication type (i.e., study protocol, conference proceeding, clinical study, retrospective study, literature review, commentary, withdrawal, older version of same paper)	Alkthouki et al., 2020; Attari et al., 2006; Bücher et al., 2015; Burgess et al., 2002; Chadwick, 2001; Cheung et al., 1990; Croll et al., 2002; Curzon & Toumba, 2006; de Gee et al., 1996; Demarco et al., 2012; Dhar et al., 2015; Espelid et al., 1999; Finucane et al., 2019; Frencken et al., 2012a; Frencken et al., 2012b; Full et al., 2013; Gao et al., 2018; Guelmann et al., 2011; Hamouda et al., 2021; Heintze et al., 2015; Hickel et al., 2005; Hilgert et al., 2014; Kilpatrick et al., 1993; Kopel et al., 1997; Krämer et al., 2007a; Krämer et al., 2007b; Levey et al., 2017; Ludovichetti et al., 2021; Manhart et al., 2004; Marks et al., 2010; McComb et al., 2001; Mickenautsch et al., 2009; Mickenautsch et al., 2012; Mjör & Gordan, 1999; Randall et al., 2002; Ricketts et al., 2006; Saber et al., 2019; Santamaria et al., 2020; Schmoeckel et al., 2020; Schwendicke et al., 2017; Seale et al., 2002; Seale et al., 2015; Sidhu & Nicholson, 2016; Smales et al., 2000; Tyas et al., 2000; Waggoner et al., 2002; Waggoner 2015; Yengopal et al., 2009; Yip et al., 2001; Chadwick et al., 2017; Raggio et al., 2013; Foley et al., 2006.	52
Full-text not retrieved	Jonnes et al., 1999; Kielbassa et al., 2016; Magnusson et al., 1976; Pettar et al., 2011; Santamaria et al., 2018; Schulte et al., 1999; Smales et al., 2002; van de Sande et al., 2016; van Waes et al., 1993.	9
Total		94

Table S3. Citation matrix for the primary studies included in the reviews of the current umbrella review.

**Review<br>Primary Studies**	**Siokis et al., 2021**	**Chisini et al. 2018**	**Delgado et al., 2021**	**Dias et al., 2018**	**Frencken et al., 2021**	**Kilpatrick et al., 2007**	**Mickenautsch et al., 2010**	**Mickenautsch et al., 2011**	**Ruengrungsom et al., 2018**	**Tedesco et al., 2017**	**Tedesco et al., 2018**	**Pires et al., 2018**	**Van t¨Hof et al., 2006**	**Innes et al., 2015**
Abo-Hamar et al., 1992									X					
Abo-Hamar et al., 2015		X												
Andersson et al., 1995		X		X										
Andersson-Wenckert et al., 2006	X								X			X		
Attabek et al., 2017			X											
Attin et al., 2001		X												
Atieh et al., 2008														X
Barr-Agholme et al., 1991						X						X		
Borges et al., 2012											X			
Bonifacio et al., 2013									X					
Buecher et al., 2015		X												
Casagrande et al., 2013	X			X								X		
Cavaheiro et al., 2020			X											
Croll et al., 2001		X							X					
Daou et al., 2009								X						
de Amorim et al., 2014		X							X					
de Franka et al., 2011									X					
Dermata et al., 2018	X													

Donly et al., 1999						X			X					
Donmez et al., 2016	X		X	X					X			X		
Dunagthip et al., 2016											X			
Duggal et al., 2002		X				X						X		
Dutta et al., 2001												X		
Ersin et al., 2006				X	X				X	X	X			
Ersin et al., 2008									X					
Espelid et al., 1999		X							X					
Frencken et al., 1994													X	
Folkesson et al., 1999		X							X					
Franzon et al., 2015		X												
Frencken et al., 2006							X							
Frencken et al., 2007							X							
Fuks et al., 2000				X		X			X					
Gao et al., 2003							X							
Giannettei et al., 2018			X											
Gross et al., 2001		X												
Hesse et al., 2014											X			
Hesse et al., 2016a									X					
Hesse et al., 2016b									X					
Hilgert et al., 2014					X				X	X				
Holst et al., 1996		X												
Honkala et al., 2003					X	X	X		X	X	X		X	
Hubel & Mejare 2003	X	X							X			X		
Hutcheson et al., 2012														X
Hse-wei et al., 1997												X		
Innes et al., 2011											X			X

Kavvadia et al., 2004		X				X						X	
Kemoli et al., 2011									X				
Kemoli et al., 2014									X				
Kilpatrick et al., 1995		X							X				
Kitty & Wei, 1997		X											
Kotsanos & Arizos, 2011		X											
Kotsanos & Dionysopoulos 2004						X							
Kraemer & Frankenberger, 2001		X							X				
Leith & O'Connell, 2011		X											
Lenzi et al., 2017			X										
Lo et al., 2001									X				
Lo & Holmgren, 2001													X
Louw et al., 2002											X		X
Luo et al., 1999													X
Marks et al., 1999						X						X	
Mass et al., 1999						X							
Mijan et al., 2014											X		
Molina et al., 2018					X				X				
Oldenburg et al., 1987						X						X	
Ostlund et al., 1992	X			X		X		X	X				
Oter et al., 2018			X										
Pascon et al., 2006												X	
Papagiannoulis et al., 1999		X											
Pereira et al., 2002				X									
Peters et al., 1996		X											

Pinto et al., 2014		X												
Qvist et al., 1997						X		X	X					
Qvist et al., 2004						X			X					
Ram et al., 2003														X
Roberts et al., 2005		X									X			
Rutar et al., 2000		X												
Rutar et al., 2002									X					
Sabbagh et al., 2017			X											
Santamaria et al., 2014											X			X
Santos et al., 2009														
Santos et al., 2010	X			X					X			X		
Santos et al., 2012											X			
Schueler et al., 2014		X												
Sengul et al., 2015	X	X		X					X			X		
Serin et al., 2019			X											
Serpa et al., 2016				X										
Sonicini et al., 2007		X												
Taifour et al., 2002		X			X	X	X	X	X	X	X		X	
Tal et al., 2017									X					
Tonn et al., 1980						X						X		
Van de Dungen et al., 2004											X			
Van Gemert et al., 2007									X					
Walls et al., 1988						X								
Webman et al., 2016		X							X					
Welbury et al., 1991						X		X				X		
Welbury et al., 2000	X											X		
Yee et al., 2001									X				X	

Yip et al., 2002			X						X
Yip et al., 2001									X
Yu et al., 2004	X		X	X	X		X		X
Zhi et al., 2012							X		
